# Leisure-time physical activity motives and perceived gains for individuals with spinal cord injury

**DOI:** 10.1038/s41393-024-01013-5

**Published:** 2024-07-30

**Authors:** Paul K. Watson, Laura Stendell, Camila Quel de Oliveira, James W. Middleton, Mohit Arora, Glen M. Davis

**Affiliations:** 1 Discipline of Exercise and Sport Sciences, Sydney School of Health Sciences, Faulty of Medicine and Health, The University of Sydney, Sydney, NSW Australia; 2 Discipline of Physiotherapy, Graduate School of Health, Faculty of Health, University of Technology Sydney, Sydney, NSW Australia; 3 John Walsh Centre for Rehabilitation Research, Northern Sydney Local Health District, St Leonards, Sydney, NSW Australia; 4 Kolling Institute, Faculty of Medicine and Health, The University of Sydney, Sydney, NSW Australia

**Keywords:** Health care, Health services

## Abstract

**Study design:**

Longitudinal cross-sectional.

**Objectives:**

To examine motives to, and perceived gains from, leisure-time physical activity (LTPA) in people with spinal cord injury (SCI).

**Setting:**

Community.

**Methods:**

One hundred and five physically active individuals with SCI undertook an online survey and a semi-structured interview. The Exercise Motives and Gains Inventory was used to examine the movies towards, and the gains from LTPA, and the Leisure-time Physical Activity Questionnaire was administered via interview to gather LTPA data. A cross-sectional analysis, which included descriptive, inferential, and regression statistics, was conducted on all participants, physical activity (PA) guideline adherers and PA guideline non-adherers.

**Results:**

The most common motives for LTPA were improvements in health and fitness, management of appearance and weight, and avoidance of illness. The most common gains from LTPA included improved health, fitness, strength and endurance, increased nimbleness, and enjoyment and revitalisation.

**Conclusions:**

Whilst health enhancement appears to be a significant motivator for LTPA, other psychosocial aspects, such as affiliation and revitalisation, appear to influence engagement and volume of LTPA. Regular LTPA should be encouraged for its health benefits, and emphasis should be placed on promoting its ability to reduce illness, facilitate affiliation, and manage stress.

## Introduction

Beyond the immediate disruption of neural communication leading to paralysis or paresis, individuals who suffer a spinal cord injury (SCI) often experience a cascade of secondary health complications. These include respiratory compromise, cardiovascular illness, and bladder and bowel dysfunction [1, 2]. Chronic pain and mental health disorders, such as depression and anxiety, are frequently reported in this population, underscoring the complex interaction between physical and psychological health challenges after SCI [3].

Leisure-time physical activity (LTPA) is a subsect of physical activity (PA) and is an activity people choose to do in their free time, such as sports, exercise, or going for a walk/wheel. Regular engagement in LTPA can lead to numerous physiological, psychological, and functional benefits for people with SCI. Physiologically, LTPA can help improve cardiovascular and metabolic health, reduce the risk of secondary complications, enhance respiratory function, and decrease hospitalisation rates [4]. Some evidence has suggested that regular engagement in task-specific exercises can lead to neuroplastic changes that might promote functional recovery after SCI [5]. Psychologically, regular LTPA participation has been associated with decreased depressive symptoms, increased self-esteem, and improved life satisfaction [6]. Furthermore, participation in adapted sports or exercise can bolster social inclusion, provide a sense of community, and improve quality of life for those with SCI [7].

Research has shown that the SCI population is relatively sedentary [8, 9], numerous barriers to LTPA exist [10], and only 10–20% of people meet SCI-specific PA guidelines [8, 9]. These guidelines have recommended that people with SCI should undertake at least two sessions of 20 min of aerobic PA (at moderate-to-vigorous intensity) and three sessions of strength exercises (for major muscle groups) twice weekly for fitness benefits to accrue [11]. People who exceed these guidelines can achieve cardiometabolic health improvements [11].

The relationship between motivation and engagement in LTPA is complex and has garnered significant attention in health and exercise psychology. Intrinsic motivation, characterised by engaging in an activity for its inherent satisfaction and enjoyment, has been linked to longer-term adherence to LTPA regimens [12]. Intrinsic motivators, such as the desire for enhanced physical health, psychological well-being, and increased social integration, are critical determinants and potent drivers of exercise adherence. Conversely, external rewards or pressures might be adequate incentives for initiating LTPA but often lack sustainability over time [13].

Scelza et al. [13]. reported that individuals with SCI who displayed intrinsic motivation towards exercising were more likely to be consistently active than those motivated by external factors. However, external factors, including access to specialised equipment, exercise support by experienced service providers, and social support, have been identified as significant facilitators for PA uptake and long-term adherence in people with SCI [14]. It appears that both intrinsic and extrinsic factors motivate people with SCI to undertake LTPA.

Given that LTPA volume is relatively poor in the SCI population, understanding the motives and perceived gains of engaging in LTPA may assist in improving participation. Hence, the primary aim of this study was to investigate motivational factors and the perceived benefits of regular engagement in LTPA for individuals with SCI. The secondary aims of the study were to examine how motives and perceived gains differed between SCI-specific PA guideline adherers and non-adherers and to determine if there were any relationships between the motives and gains of LTPA with the volume of LTPA performed. We hypothesised that motivation to engage in LTPA would be driven primarily by improvement to health, and the most notable gains would pertain to avoidance of ill health and injury, as well as an improvement in fitness and appearance.

## Methods

### Design and recruitment

This prospective cross-sectional study enrolled 105 participants with SCI from across Australia. Recruitment was primarily through SCI-specific consumer and support organisations. Recruitment methods involved social media advertisements, generic emailing, and ‘snowball’ sampling. The survey commenced in September 2022 and concluded in July 2023.

The survey was available via the Research Electronic Data Capture (REDCap) software (https://www.project-redcap.org/), an online survey and database management platform. To facilitate access, all promotional materials, such as emails and advertisements, featured a quick response code and a direct link to the survey. Participants who preferred other methods to complete the survey could contact the research team to ask for a hard copy of the survey questions to be mailed. Before accessing the survey, participants had to review and acknowledge a Participant Information Statement. Completing the survey was voluntary, and participants were offered an electronic gift card for participating.

Participants independently completed the demographic and motives and gains surveys online. Semi-structured interviews, conducted online or via telephone, were used to gather data on LTPA volume. The interviews were conducted after participants autonomously completed the online demographic and motive and gains surveys. Two study investigators facilitated the interviews, one with accreditation as an Exercise Physiologist and the other registered with the Australian Health Practitioner Regulation Agency as a Physiotherapist. Both investigators had prior experience working with individuals with SCI and administering survey instruments. Before commencing the interviews, participants were provided with a comprehensive definition of LTPA and instructed to contemplate LTPA specifically while responding to the motives and gains survey.

### Participants

To qualify for inclusion, participants needed to be 18 years or older, live in Australia, be at least 12 months post-injury, and have a diagnosed SCI from either a traumatic event or a non-traumatic disorder. Participants needed to be engaged in some volume of LTPA in the previous seven days, which was confirmed in the interview component of the study by the study investigators. Individuals with compromised cognitive abilities, those needing intensive medical care due to unstable health conditions, or those who had undergone spinal surgery within the last 30 days were omitted.

### Data collection

Information on sociodemographic factors and SCI characteristics was gathered using a questionnaire custom-made for this study but based on earlier population-level research in this domain [15].

The Leisure Time Physical Activity Questionnaire for Individuals with Spinal Cord Injury (LTPAQ-SCI) [16] was utilised to gauge the volume of participation in LTPA. This recall tool evaluates time spent in mild, moderate, and vigorous-intensity aerobic LTPA and strength-related exercises undertaken in the past seven days. The LTPAQ-SCI is reliable and accurate in gauging LTPA participation in those with SCI [16].

To facilitate precise recall of LTPA among study participants, two researchers, the first and senior author, developed an exercise intensity infographic (Supplement 1). The infographic illustrated the continuum of LTPA intensity by using exemplars of associated activities and quantitative metrics, including heart rate, breathing rate, and perceived energy expenditure. The infographic was provided to participants within the survey portal in REDCap, and all participants in the study acknowledged reading and understanding it before being interviewed.

Motives and gains data was captured using the Exercise Motives and Gains Inventory (EMGI) [17]. This questionnaire measured motives and perceived gains from engaging in PA and was developed based on the Exercise Motivations Inventory 2 (EMI-2) [18]. The EMI-2 is a valid and reliable tool to examine the motivation for PA [17] and has been used previously in the SCI population [19]. By adding a gains construct to the motive paradigm of the EMI-2, the EMGI provided a means of determining perceived gains achieved through exercise. The correlation between the motive and gain constructs within the EMGI has been validated [17].

The EMGI survey consists of 51 motives and 51 gains four-point scale questions. The data gathered from the EMGI pertains to the participants’ motives toward LTPA and the gains they experienced from LTPA at the time of completing the survey. During analysis, these questions are grouped (usually four questions per group) into 14 motive and 14 gain subscales [17]: social recognition, enjoyment, challenge, competition, affiliation, revitalisation, health pressures, ill-health avoidance, positive health, weight management, stress management, appearance, strength and endurance, and nimbleness. These 14 motive/gain subscales are then narrowed into five categories [17]: social engagement (enjoyment, revitalisation, and stress management); adverse health (health pressures and ill-health avoidance); health/fitness (positive health, strength/endurance, nimbleness, and ill-health avoidance); appearance/weight management (as a motive); and weight management (as a gain, primarily associated with health and fitness).

### Data analysis

Statistical analyses were performed with SPSS version 28 (IBM SPSS, Inc., Chicago, IL). Descriptive statistics were calculated for both sociodemographic and injury characteristics. Additionally, each participant’s average weekly minutes (min/wk) of participation in LTPA was determined, and the averages for mild, moderate, vigorous, moderate-to-vigorous intensity LTPA, total LTPA and strength LTPA categories were calculated. Compliance with PA guidelines was assessed based on the SCI-specific PA guidelines [11].

Motives and perceived gains were examined in three groups: all participants, PA guideline-adherers, and PA guideline non-adherers. The mean and standard deviation for each category and subscale of the EMGI were then calculated. T-tests were conducted between (i) the motive and gain score for each category and subscale of the EMGI and (ii) the score of the five EMGI categories and 14 subscales between PA guideline-adherers and non-adherers.

Regression modelling was utilised to investigate the relationship between (i) the motive categories and the volumes of LTPA performed and (ii) the amount of LTPA performed and the reported gains participants experienced. A 10,000 bootstrap was used for regression modelling.

## Results

### Participant characteristics

One hundred and five Australians with SCI who self-reported as people who undertook regular LTPA participated in the survey. Of these, 66 (63%) were males. The mean (SD) age of the participants was 56 (15) years, and on average, they had lived with their injury for 13 (14) years. Out of the total, 61 participants (58%) had paraplegia, 73% had an incomplete injury, and the majority (81%) had a traumatic injury. Participants in our study resembled those of the broader Australian SCI population, where 73% of people with SCI are male, have a mean (SD) age of 57 (14) years, and average time since injury of 17 (14) years [20]. Table 1 provides data on the sociodemographic and injury characteristics of the included participants.

**Table 1:** Sociodemographic and injury characteristics of participants.

Age at the time of survey (years)	Mean (SD)
Time since injury (years)	
	56.1 (14.9) 13.2 (13.6)
	**n (%)**
Gender	
Male	66 (62.9)
Female	39 (37.1)
Relationship status	
Single	50 (47.6)
Partnership	54 (47.6)
Missing	1 (1.0)
Household income	
Below-average household income	64 (61.0)
Above-average household income	29 (27.6)
Missing	12 (11.4)
Highest educational level	
Primary to Secondary	25 (23.8)
Post-secondary or Tertiary	30 (28.6)
Bachelor or Masters	44 (41.9)
PhD	6 (5.7)
Injury level	
Paraplegia	51 (58.1)
Tetraplegia	44 (41.9)
Completeness	
Complete	20 (28.6)
Incomplete	73 (69.5)
Missing	12 (11.4)
Cause of injury	
Traumatic	81 (77.1)
Non-traumatic	22 (21.0)
Missing	12 (11.4)
Employed	
No	37 (35.2)
Yes	43 (41.0)
Missing	25 (23.8)
Ambulation less than 100 m	
Manual wheelchair	35 (33.3)
Electric wheelchair/scooter	13 (12.4)
Walking device(s)	15 (14.3)
No assistive device(s)	8 (7.6)
Missing	34 (32.4)
Ambulation of more than 100 m	
Manual wheelchair	33 (31.4)
Electric wheelchair/scooter	27 (25.7)
Walking device(s)	7 (6.7)
No assistive device(s)	4 (3.8)
Missing	34 (32.4)

### Leisure-time physical activity and PA guideline compliance

On average (SD), participants engaged in 301 (257) min/wk of total LTPA, with a median of 210 min/wk. The mean participation in moderate-to-vigorous LTPA (MV-LTPA) was 123 (165) min/wk, and its median was 80 min/wk. The average amount of strength LTPA was 96 (115) min/wk with a median of 60 min. The highest reported total LTPA was 1410 min/wk, while the lowest was 23 min/wk. Approximately half of the participants (47%) adhered to the minimum SCI-specific PA guideline recommendations. Data on participation in LTPA and PA guideline compliance can be found in Table 2.

**Table 2:** Descriptive statistics of leisure-time physical activity.

LTPA variable	Mean (SD) (min/wk)	Median (min/wk)	Range (min/wk)
Mild LTPA	107 (154)	70	5–1120
Moderate LTPA	96 (130)	60	5–720
Vigorous LTPA	26 (57)	0	10–300
Strength LTPA	96 (115)	60	10–660
MV-LTPA	123 (165)	80	5–990
Total LTPA	301 (257)	210	23–1410

Total PA guideline compliance - Achieved: *n* = 49 (47%), Not achieved: *n* = 56 (53%)

PA guideline compliance is measured against the minimum recommended PA for fitness benefits [11] in the PA guideline recommendations

*PA* physical activity, *min/wk* minutes per week, *LTPA* leisure-time physical activity, *MV-LTPA* moderate-to-vigorous LTPA

### Motives and gains

**Fig. 1:**
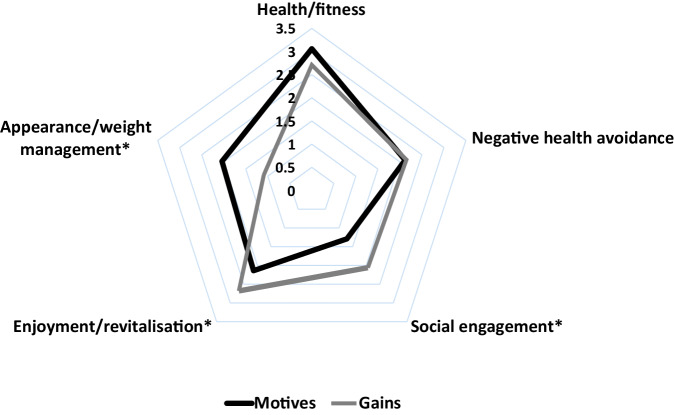
Motive and gain category scores for all participants. *Scores between motives and gains were significantly different at the *p* ≤ 0.05 level. Each motive/gain item had a maximum score of 4.

**Fig. 2:**
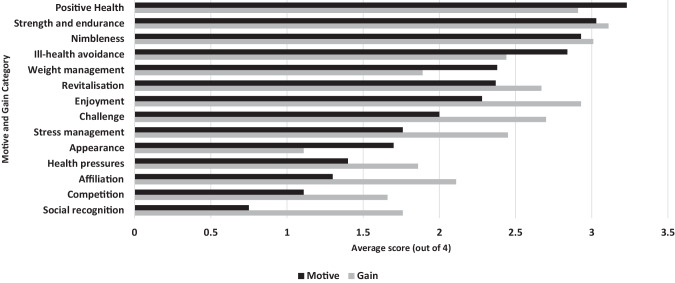
Motive and gain subscale scores for all participants.

Table 3 and Figs. 1 and 2 present the results of the EMGI analysis. Cochrane’s alpha for the internal consistency of each of the 14 EMGI subscales was above 0.95 for all motives questions and 0.98 for all gains questions, suggesting accuracy between the four questions grouped to make each subscale.

**Table 3:** Motives and gains of leisure-time physical activity.

	All participants		Guideline-adherers		Guideline non-adherers	
	Motivation	Gain	Motivation	Gain	Motivation	Gain
	Categories					
Social engagement	1.29 (1.35)*	2.06 (1.34)*	1.51 (1.41)	2.05 (1.24)	1.21 (1.32)	1.91 (1.39)
Negative health avoidance	2.12 (1.27)	2.15 (1.27)	2.26 (1.26)	1.72 (1.14)	2.05 (1.15)	1.94 (1.31)
Health/Fitness	3.06 (1.04)	2.71 (1.10)	3.31 (0.91)	3.04 (0.83)**	2.99 (1.00)	2.49 (1.20)**
Enjoyment/revitalisation	2.14 (1.37)*	2.68 (1.20)*	2.51 (1.17)	3.06 (0.98)**	2.13 (1.34)	2.46 (1.26)**
Appearance/weight management	2.04 (1.04)*	–	1.98 (1.29)	–	2.05 (1.46)	–
Weight management	–	1.09 (0.81)*	–	1.93 (1.07)	–	1.90 (1.31)
	Subscales					
Social recognition	0.75 (1.21)*	1.76 (1.35)*	0.89 (1.32)	1.97 (1.31)	0.68 (1.15)	1.66 (1.38)
Enjoyment	2.28 (1.42)*	2.93 (1.17)*	2.74 (1.18)	3.34 (0.84)**	2.28 (1.12)	2.69 (1.27)**
Challenge	2.00 (1.41)*	2.70 (1.18)*	2.34 (1.43)	2.99 (1.01)	1.85 (1.34)	2.54 (1.23)
Competition	1.11 (1.43)	1.66 (1.47)	1.39 (1.54)	1.99 (1.39)	1.04 (1.41)	1.47 (1.50)
Affiliation	1.30 (1.33)*	2.11 (1.36)*	1.40 (1.38)	2.34 (1.23)	1.26 (1.36)	1.98 (1.45)
Revitalisation	2.37 (1.32)	2.67 (1.20)	2.76 (1.07)	3.09 (1.00)**	2.35 (1.44)	2.41 (1.23)**
Health pressures	1.40 (1.32)	1.86 (1.38)	1.51 (1.28)	2.12 (1.31)	1.27 (1.32)	1.66 (1.38)
Ill-health avoidance	2.84 (1.21)	2.44 (1.16)	3.01 (1.24)	2.77 (0.97)	2.82 (0.98)	2.22 (1.24)
Positive Health	3.23 (0.93)	2.91 (1.08)	3.43 (0.89)	3.24 (0.86)**	3.20 (0.73)	2.68 (1.16)**
Weight management	2.38 (1.40)	1.89 (1.23)	2.26 (1.37)	2.37 (1.07)	2.50 (1.43)	1.90 (1.31)
Stress management	1.76 (1.37)*	2.45 (1.23)*	2.04 (1.27)	2.76 (1.11)	1.76 (1.46)	2.27 (1.27)
Appearance	1.70 (1.35)*	1.11 (1.25)*	1.70 (1.20)	2.03 (1.15)	1.60 (1.48)	1.66 (1.32)
Strength and endurance	3.03 (1.08)	3.01 (1.04)	3.34 (0.86)	3.57 (0.62)**	2.93 (1.14)	2.82 (1.12)**
Nimbleness	2.93 (1.11)	3.11 (1.02)	3.16 (0.98)	3.30 (0.67)**	2.84 (1.14)	2.80 (1.20)**

Scores under the categories and subscales are mean (SD)

The minimum score is 0, and the maximum score is 4

The missing values in the Categories section are explained in the instrument scoring instructions [17]. The Appearance motive in the categories is captured within the Appearance/weight motive category, whereas the Appearance gain in the categories is captured within the Health/fitness category. These two Categories were compared to each other in the t-test analysis.

*p ≤ 0.05 in dependent t-test between motivation and gain score for all participants; **p ≤ 0.05 in independent t-test between motive and gain score for guideline adherers and guideline non-adherers.

Within the EMGI subscale scores, a desire for positive health (3.23), improvement in strength and endurance (3.03), and furtherance of nimbleness (2.93) were the most common motives, and improved strength and endurance (3.11), enjoyment (2.93), and nimbleness (3.01) were the highest report gains of regular LTPA. Participants who were non-adherent to PA guidelines reported lower motives and gains subscale scores than individuals who reported LTPA volumes that met PA guideline recommendations. The motives of enjoyment, health improvement, strength and endurance advancements, and development of nimbleness were significantly higher (*p* < 0.05) in PA guideline-adherers compared to non-adherers.

In the EMGI categories, social engagement was the lowest motive (1.29), and weight management was the lowest reported gain (1.09) for LTPA participation. Participants reported significantly higher gain scores in social recognition, enjoyment, affiliation, and stress management (*p* < 0.05) than motive scores. However, gains scores of improved appearance and weight management were significantly lower than motive scores (*p* < 0.05).

Enjoyment/revitalisation and health and fitness were the only motives significantly associated with participation in MV-LTPA (*p* = 0.005) and total LTPA (*p* = 0.05). Despite model significance [R^2^ = 0.21, F(5, 63) = 6.71, *p* = 0.009], no significant predictor variables were found in the MV-LTPA multivariate analysis.

Perceived gains in appearance and in the ability to manage weight were significantly associated [*R*^2^ = 0.12, F(4, 74) = 6.71, *p* = 0.05] with the volume of total LTPA (*p* = 0.031) and mild LTPA (*p* = 0.008) performed. Health and fitness benefits were positively associated with the total volume of LTPA performed [*R*^2^ = 0.19, F(4, 73) = 6.71, *p* = 0.004]. Still, no single intensity of LTPA was more influential in generating improvements in appearance and weight management. Multivariate regression analyses for motives predicting LTPA are available in Supplement 2, and regression analysis of perceived gains from LTPA is available in Supplement 3.

## Discussion

This exploratory study investigated motives to, and perceived gains from LTPA in people with SCI. Motives of LTPA were primarily associated with improving health and fitness. Improvements in strength, endurance, and nimbleness were often reported as perceived gains of LTPA participation. Gains scores were higher than motive scores in social recognition, affiliation, and enjoyment. Despite being motivated to engage in LTPA for appearance and weight improvement, participants reported lower gain scores in these areas. Participants who adhered to the SCI-specific PA guidelines reported significantly more benefits to their health, fitness, and nimbleness, and more significant ill-health avoidance than those who did not meet the guidelines.

Usually, the LTPAQ-SCI is administered to study participants in the form of a self-administered questionnaire. Using interviews allowed the investigators to examine and scrutinise the LTPA disclosed by study participants. Intriguingly, some subjects failed to categorise their engagement in exercises conducted at rehabilitation clinics as instances of LTPA despite providing accounts of activities that met the criteria for LTPA according to the investigators’ assessment. These individuals considered rehabilitation activities as discretely different from LTPA. Thus, using interview-based data collection methods proved valuable, enabling investigators to clarify participant responses and aiding in the accuracy and comprehensiveness of the LTPA data.

Participants ranked health, fitness, and nimbleness enhancements as their primary motives and perceived gains for undertaking LTPA. This finding is consistent with prior research [19] that utilised the EMI-2 to investigate the motivators for PA in individuals with paraplegia due to an SCI. Research has shown that LTPA may improve voluntary movement, physical capacity, and energy expenditure, which in turn can lead to improvements in the health of the cardiorespiratory, metabolic, and musculoskeletal systems [21]. Furthermore, enhancing strength and stamina can bolster autonomy, augment ability and decision-making, and minimise the risk of musculoskeletal overuse injuries [22]. Based on these findings, with those of this study, we recommend facilitating LTPA from the earliest stages of SCI rehabilitation and throughout later community life.

Our findings revealed that the average gains score was significantly higher than the average motive score for enjoyment and revitalisation through LTPA. This may suggest that individuals derived more pleasure and felt more revitalised by LTPA than initially anticipated. Enjoyment is essential to maintaining positive long-term behaviour toward LTPA [23]. Barriers to LTPA reported in this population, such as laziness, lack of internal motivation, and perceived lack of return on time investment, can be mitigated by LTPA, which participants enjoy [13]. Similarly, people in this study were reasonably motivated to change their appearance and improve their weight through LTPA. Yet, perceived gains scores in this category were significantly lower than motive scores, suggesting that participants may not have achieved the change to their weight and appearance that they had hoped. Post-injury metabolic changes can reduce energy expenditure, often resulting in weight gain [24] in people with SCI. In addition, limited mobility and muscle atrophy can reduce the calories expended daily. This means that traditional diet and exercise recommendations regarding weight loss may not be practical in this population. Thus, tailored dietary strategies focusing on reducing calorie intake while ensuring optimal nutrient density may be crucial for people with SCI. Our results corroborate previous study recommendations, which suggested that PA and diet modification should be considered part of PA interventions [24], as LTPA alone may not elicit adequate benefits to appearance and weight for people with SCI.

The motive categories of the EMGI only slightly predicted the volume of LTPA reported (21% and 25% variance of the MV-LTPA and total LTPA regression models, respectively). Similarly, the volume of LTPA performed only very slightly predicted the gains examined in the tool (12% and 19% of the variance in the MV-LTPA and total LTPA regression models, respectively). Practical tasks such as setting goals and monitoring progress lead to the perceptible enhancement of one’s physical capabilities and can create a positive feedback loop, bolstering an individual’s intrinsic motivation to persist with exercise routines [25]. When individuals can see, feel, and measure their progress, the abstract benefits of exercise become tangible, making the pursuit of health and fitness an even more compelling motivating factor. Therefore, it is unsurprising that health improvements and strength and endurance enhancement were the most potent motives and highest reported gains of LTPA for participants in our study. Behavioural frameworks such as the Self-Determination and the Social Cognitive Theory posit that intrinsic motivation and self-efficacy are crucial for behaviour adoption and long-term compliance. Tailoring exercise programs, establishing goals, providing positive feedback, and facilitating mastery experiences can significantly increase LTPA volumes for people with SCI [26]. Service providers should closely monitor and acknowledge client LTPA and fitness achievements (including adherence) while emphasising the great potential of regular LTPA to manage comorbidity, improve health, and provide enjoyment and affiliation to clients to drive LTPA motivation.

Social recognition, affiliation, and stress management gain scores were significantly higher than equivalent motive scores. A systematic review [27] has provided comprehensive insights into the positive impact of social connectedness on various aspects such as resilience, adaptability, health, and functional status for people with SCI. The cultivation of social networks through engagement in recreational and sporting activities, complemented by interactions with individuals who share similar life experiences, has been identified as a critical driver for enhanced involvement in PA [27]. Additionally, the emotional adjustment after SCI is profound, and new psychosocial stressors emerge rapidly after injury [28]. PA is an established way to mitigate these emergent stressors, and individuals who report greater satisfaction with social support are known to be more satisfied with life and health than those with less affiliation [28]. Allied health practitioners should, therefore, ensure that social connectedness and recognition are facilitated in community exercise programs and encourage LTPA uptake for the psychosocial improvements demonstrated by our findings.

Data herein could be used to enhance LTPA volume for individuals not currently achieving PA guideline recommendations. Our results indicated that people who completed the recommended [11] dose (i.e., performed more min/week) of LTPA felt more revitalised and reported greater overall health. A greater (self-reported) improvement in health by those who relayed more LTPA is unsurprising. Hoevenaars et al. [29]. showed that meeting exercise guidelines was associated with better respiratory function, exercise capacity, fitness and (some) body composition outcomes in the SCI population. Research has also suggested that educating exercisers to self-regulate their intensity (so that individuals autonomously exercise near ventilatory or lactic threshold) fosters a greater tolerance and sense of pleasure toward PA, which in turn may improve volumes performed [30]. Service providers should ensure that even those not amenable to higher activity volumes have a wide variety of exercises to undertake and are allowed a degree of autonomy with choice in physical engagement and exercise intensity. These strategies may improve the enjoyment of LTPA, and a higher degree of enjoyment has been linked to greater volumes of PA [23].

### Limitations

When interpreting these findings, consideration should be given to the limited sample size (*n* = 105). A larger sample would be required to achieve sufficient statistical power. Also, future research should expand the range of motive and gain constructs examined, and utilise qualitative methodologies to further explore factors that could influence motives toward and perceived gains from LTPA for people with SCI.

Data extraction may have been influenced by the different interview styles of the two study investigators who administered the interviews. Investigators attempted to mitigate this by strictly adhering to survey instructions and interview scripts. Also, self-reports of LTPA can be influenced by social desirability and approval [31], which may inflate LTPA volumes. The study investigators conducting the interviews tried to avoid prompting when gathering LTPA data to manage this. Finally, given that our sample was solely from within Australia, we recognise that our results may not represent individuals with SCI from other countries, especially those from different socio-economic or ethnoreligious regions where the motives and barriers to LTPA may vary.

## Conclusion

The desire to improve one’s health and fitness and manage appearance were the primary motives for people with SCI to engage in LTPA. Whilst benefits in these areas were reported, gains or improvements in these areas appear to be low, and gains in affiliation and stress management are reported more often. Motives between PA guideline-adherers and non-adherers did not differ significantly, except in the desire to avoid ill health. Still, guideline-adherers reported more enjoyment, better overall health, and greater fitness from LTPA than those who did not meet PA guidelines. Regular LTPA should be encouraged for its health and fitness benefits, and emphasis should be placed on promoting its ability to prevent illness, facilitate affiliation and manage stress in the SCI population.

### Supplementary information

Supplementary material

## Data Availability

Data is available upon request from the authors.
