# Supplementary material for: Leisure-time physical activity motives and perceived gains for individuals with spinal cord injury
Source: Spinal Cord. 2024 Jul 30;62(9):546–52. doi: 10.1038/s41393-024-01013-5 (PMC11368805; doi:10.1038/s41393-024-01013-5)
Supplement: Supplementary file 1 — Supplementary material [file 41393_2024_1013_MOESM1_ESM.docx]

**Supplement 1. Physical activity infographic**

**
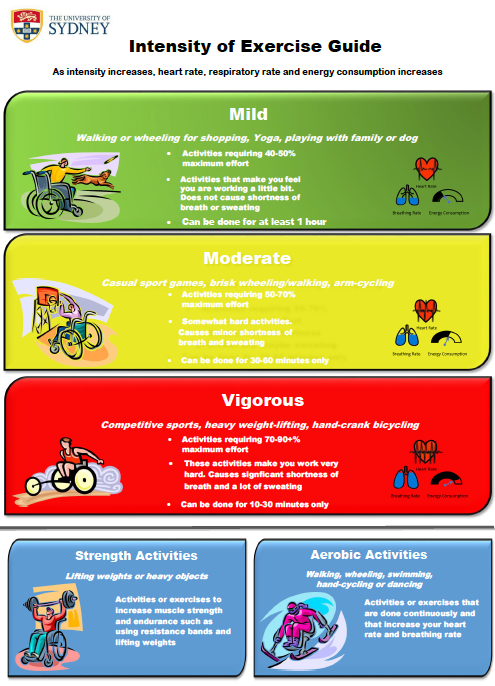
**

**Supplement 2. Multivariate regression of motivations predicting leisure-time physical activity volume**

| **LTPA** | **Predicting motive** | **B** | **s.e.** | **β** | **t** | **p-value** |
| --- | --- | --- | --- | --- | --- | --- |
| Mild  R^2^ = 0.06  p = .553 | Appearance/weight management | -1.57 | 3.68 | -0.06 | -0.37 | .674 |
|  | Social engagement | -3.00 | 3.17 | -0.26 | -1.36 | .399 |
|  | Enjoyment/revitalisation | 4.22 | 3.67 | 0.31 | 1.46 | .295 |
|  | Negative health avoidance | -0.89 | 8.72 | -0.02 | -0.12 | .915 |
|  | Health and fitness | 2.46 | 6.4 | 0.09 | 0.50 | .738 |
| MV  R^2^ = 0.21  **p = .009** | Appearance/weight management | -7.15 | 4.05 | -0.27 | -1.77 | .082 |
|  | Social engagement | 3.08 | 2.08 | 0.26 | 1.48 | .143 |
|  | Enjoyment/revitalisation | 2.63 | 2.73 | 0.19 | 0.97 | .338 |
|  | Negative health avoidance | -3 | 7.21 | -0.07 | -0.41 | .683 |
|  | Health and fitness | 7.26 | 4.65 | 0.26 | 0.12 | .123 |
| Strength  R^2^ = 0.10  p = .258 | Appearance/weight management | -3.53 | 2.43 | -0.24 | -1.45 | .151 |
|  | Social engagement | 0.31 | 1.25 | 0.05 | 0.25 | .805 |
|  | Enjoyment/revitalisation | 0.92 | 1.64 | 0.12 | 0.56 | .577 |
|  | Negative health avoidance | -3.82 | 4.32 | -0.17 | -0.88 | .380 |
|  | Health and fitness | 4.74 | 2.79 | 0.3 | 1.70 | .094 |
| Total  R^2^ = 0.25  **p = .002** | Appearance/weight management | -10.28 | 6.13 | -0.25 | -1.68 | **.050** |
|  | Social engagement | -0.19 | 3.14 | -0.01 | -0.06 | .963 |
|  | Enjoyment/revitalisation | 8.62 | 4.13 | 0.4 | 2.09 | .089 |
|  | Negative health avoidance | -12.08 | 10.91 | -0.19 | -1.12 | .228 |
|  | Health and fitness | 15.88 | 7.04 | 0.37 | 2.26 | **.022** |

LTPA: leisure-time physical activity; MV: moderate-to-vigorous

**Supplement 3. Multivariate regression of leisure-time physical activity volume predicting gains**

| **Gain** | **Predicting LTPA volume** | **B** | **s.e.** | **β** | **t** | **p-value** |
| --- | --- | --- | --- | --- | --- | --- |
| Appearance/weight management  R^2^ = 0.12  **p = .050** | Mild | -0.01 | 0.01 | -0.38 | -0.81 | **.008** |
|  | MV | 0 | 0.01 | -0.01 | -0.12 | .961 |
|  | Strength | -0.00 | 0.01 | -0.04 | -0.11 | .779 |
|  | Total | 0.01 | 0.01 | 0.51 | 0.65 | **.031** |
| Social engagement  R^2^ = 0.09  p = .102 | Mild | -0.01 | 0.06 | -0.07 | -0.15 | .788 |
|  | MV | 0.03 | 0.06 | 0.29 | 0.60 | .232 |
|  | Strength | 0.01 | 0.07 | 0.04 | 0.13 | .881 |
|  | Total | 0.00 | 0.06 | 0.03 | 0.04 | .960 |
| Enjoyment/revitalisation  R^2^ = 0.16  **p = .012** | Mild | -0.02 | 0.03 | -0.22 | -0.48 | .188 |
|  | MV | 0.01 | 0.03 | 0.07 | 0.15 | .621 |
|  | Strength | 0.01 | 0.04 | 0.03 | 0.10 | .849 |
|  | Total | 0.02 | 0.03 | 0.44 | 0.57 | .183 |
| Negative health avoidance  R^2^ = 0.11  p = .067 | Mild | 0 | 0.02 | -0.01 | -0.02 | .970 |
|  | MV | 0.01 | 0.02 | 0.15 | 0.40 | .389 |
|  | Strength | 0.01 | 0.03 | 0.17 | 0.64 | .358 |
|  | Total | 0.00 | 0.02 | 0.1 | 0.14 | .798 |
| Health and fitness  R^2^ = 0.19  **p = .004** | Mild | -0.01 | 0.02 | -0.17 | -0.39 | .329 |
|  | MV | 0.01 | 0.02 | 0.23 | 0.51 | .202 |
|  | Strength | 0.01 | 0.03 | 0.15 | 0.50 | .465 |
|  | Total | 0.01 | 0.02 | 0.22 | 0.31 | .489 |

LTPA: leisure-time physical activity; MV: moderate-to-vigorous

**Supplement 4. Motivation and Gain scores between physical activity guideline adherents and non-adherents**

*Scores between Guideline adherents gains scores and Guideline non-adherents gains scores were significantly different at the p≤0.05 level
